# Comparison of Eco-Friendly Ionic Liquids and Commercial Bio-Derived Lubricant Additives in Terms of Tribological Performance and Aquatic Toxicity

**DOI:** 10.3390/molecules29163851

**Published:** 2024-08-14

**Authors:** Xin He, Louise M. Stevenson, Chanaka Kumara, Teresa J. Mathews, Huimin Luo, Jun Qu

**Affiliations:** 1 Materials Science & Technology Division, Oak Ridge National Laboratory, Oak Ridge, TN 37831, USA; kumarack@ornl.gov; 2 Environmental Sciences Division, Oak Ridge National Laboratory, Oak Ridge, TN 37831, USA; stevensonlm@ornl.gov (L.M.S.); mathewstj@ornl.gov (T.J.M.); 3 Manufacturing Science Division, Oak Ridge National Laboratory, Oak Ridge, TN 37831, USA; luoh@ornl.gov

**Keywords:** ionic liquids, environmentally acceptable lubricants (EALs), bio-derived lubricant additives, tribological, aquatic toxicity

## Abstract

Approximately half of the lubricants sold globally find their way into the environment. The need for Environmentally Acceptable Lubricants (EALs) is gaining increased recognition. A lubricant is composed of a base oil and multiple functional additives. The literature has been focused on EAL base oils, with much less attention given to eco-friendly additives. This study presents the tribological performance and aquatic toxicity of four short-chain phosphonium-phosphate and ammonium-phosphate ionic liquids (ILs) as candidate anti-wear and friction-reducing additives for EALs. The results are benchmarked against those of four commercial bio-derived additives. The four ILs, at a mere 0.5 wt% concentration in a synthetic ester, demonstrated a 30–40% friction reduction and >99% wear reduction, superior to the commercial baselines. More impressively, all four ILs showed significantly lower toxicity than the bio-derived products. In an EPA-standard chronic aquatic toxicity test, the sensitive model organism, Ceriodaphnia dubia, had 90–100% survival when exposed to the ILs but 0% survival in exposure to the bio-derived products at the same concentration. This study offers scientific insights for the future development of eco-friendly ILs as lubricant additives.

## 1. Introduction

The world consumes roughly 40 million tons of lubricants annually [1,2], nearly half of which eventually end up in the environment through volatilization, spills, discharges, or other losses [3]. Initial responses to lubricant leaks and spills cost more than USD 300 million annually without even counting the long-term costs [4]. Conventional lubricants are based on mineral oils and are harmful to the environment [3]. The need for moving toward Environmentally Acceptable Lubricants (EALs) is gaining increased recognition [5]. In particular, the U.S. Environmental Protection Agency (EPA) made eco-friendly lubricants mandatory for all vessels in the 2013 Vessel General Permit: “…. *every ship Operator navigating in or entering US waters MUST use Environmentally Acceptable Lubricants in all applications with oil-to-sea interface*”. The global market of EALs was USD 5.25 billion in 2023 and is expected to have a compound annual growth rate of 12.53% to reach USD 13.55 billion by 2030, which is more than triple the growth rate (3.8%) of the overall lubricants market [6].

A lubricant is composed of a base oil and various functional additives that are vital ingredients for meeting the tribological performance requirements. There are four types of EPA-approved base fluids for EALs, including water-based lubricants, vegetable oil, synthetic esters, and polyalkylene glycols [7]. Anti-wear additives, usually at a concentration of 0.5–2 wt%, play a decisive role in wear protection [8]. Conventional anti-wear additives frequently incorporate heavy metals, halogens, and/or sulfur compounds, rendering them unable to meet the toxicity or biodegradability requirements. A prime example is the widely used zinc dialkyldithiophosphate (ZDDP) [8], which was proven to be toxic in our recent report [9]. 

Ionic liquids (ILs) are frequently employed as ‘green’ solvents in various applications such as chemical synthesis, electrochemistry, and catalysis due to their ultra-low vapor pressure, non-flammability, and high thermal stability [10]. Recently, ILs have shown promising potential as ashless anti-wear and friction-reducing additives in both oil-based [11,12,13] and water-based [14,15,16] lubricants. There were some premature claims of ILs as environmentally friendly lubricants in the earlier literature without direct evidence [17,18,19]. However, our recent work clearly showed that not all ILs are benign and an IL’s toxicity depends on its chemistry and molecular structure. For example, imidazolium and pyridinium cations and halogen-containing anions have been reported to be toxic [20,21,22], and the alkyl chain length in the substituent group demonstrated a decisive role in toxicity [20,23]. A new group of ILs [24] consisting of short-chain phosphonium or ammonium cations paired with phosphate anions were invented with excellent anti-wear functionality and aquatic toxicity categorized as “Not Toxic” based on the EPA’s standard toxicity test. In particular, the ions with four-carbon alkyls have been identified as good candidates for balancing oil solubility, thermal stability, toxicity, and lubricity [9].

As a follow-on, in this study, we conducted a side-by-side comparison of the tribological behavior and toxicity of selected candidate ILs and commercial anti-wear lubricant additives. The ILs include three short-chain ammonium-phosphate ILs identified in our earlier work [9] and a newly developed short-chain phosphonium-phosphate IL. The commercial anti-wear products include one traditional ashless and four bio-derived additives. The results provide scientific insights for the future development of eco-friendly ILs as EAL additives.

## 2. Materials and Methods

### 2.1. Selection and Synthesis of ILs

Three short-chain ammonium-phosphate ILs were selected because of their superior lubricating performance and low toxicity, as observed earlier [9]. The aprotic tributyl(methyl)ammonium dibutyl phosphate, ([N_4441_][DBP]), was synthesized as follows: Tributyl(methyl)ammonium chloride (N_4441_Cl) and dibutyl phosphoric acid (HDBP) were mixed in equimolar amounts in hexane. A NaOH solution was then added dropwise to the mixture at room temperature. The mixture was stirred for 12 h at room temperature. The water phase at the bottom was separated and washed 4 times with dichloromethane (CH_2_Cl_2_). The final product was obtained by evaporating dichloromethane in a rotary evaporator. The 2 protic ammonium phosphates, tributylammonium dibutyl phosphate ([N_444_H][DBP]) and morpholine dibutyl phosphate ([Mor][DBP]), were formed by mixing an equal molar amount of the corresponding amine (N_444_ or morpholine) and dibutyl phosphoric acid (HDBP) at room temperature for 2 h through to neutralization. The liquid mixture became more viscous during stirring.

A new short-chain phosphonium-phosphate, tetrabutylphosphonium dibutyl phosphate ([P_4444_][DBP]), was developed more recently. The synthesis procedure was as follows: Tetrabutylphosphonium bromide (P_4444_Br) and dibutyl phosphoric acid (HDBP) were mixed in equimolar amounts in hexane. A NaOH solution was then added dropwise to the mixture at room temperature. The mixture was stirred for 12 h at room temperature. The water phase at the bottom was separated and washed 4 times with dichloromethane (CH_2_Cl_2_). The final product was obtained by evaporating off dichloromethane in a rotary vacuum evaporator.

The oil solubility of additives was determined by visual inspection of the oil–additive blends after 5 min of centrifuging at 13,000 rpm. The thermogravimetric analysis (TGA) was conducted on the ILs using TGA-2950 (TA Instruments, New Castle, DE, USA) in air with a 10 °C/min heating rate. 

### 2.2. Commercial Baseline Lubricant and Anti-Wear Additives

This study considered hydraulic fluids as an example application for the ILs and thus a fully formulated commercial hydraulic oil, Mobil-25 (ExxonMobil, Paulsboro, NJ, USA), was used as the baseline lubricant against which to test the performance of the candidate EALs. Five commercial anti-wear additives, a traditional ashless Irgalube^®^ 349 (BASF) and four bio-derived Additin^®^ RC3760, RC9420, RC9410, and M93.001 (Lanxess, Cologne, Germany), were selected for the side-by-side comparison with the ILs. The chemical compositions of each additive from the Safety Data Sheets are listed in Table S1.

### 2.3. Base Oils

Two base oils, BT-22 and BT-4 (Biosynthetic Technologies, Indianapolis, IN, USA), were selected as the base lubricants to evaluate the candidate anti-wear additives. These 2 base oils are synthetic esters and can be categorized as “Readily biodegradable” and “Not Toxic” based on Economic Co-operation and Development (OECD) tests, as shown in Table 1. The structures of the 2 lubricants are displayed in Figure S1 in the Supporting Information. BT-22 and BT-4 were blended into a new base oil (BT-mix) to match the viscosity grade, VG 46, of Mobil 25. The mixing ratio between the 2 BT oils was calculated based on the Refutas equation. The measured viscosities are shown in Table 1 [25].

### 2.4. Tribological Evaluation

Since a typical concentration of anti-wear additives in a hydraulic fluid is 0.5 wt%, the candidate ILs and commercial anti-wear additives were added to the BT-mix base oil at 0.5 wt% for the tribological evaluation. The tribological behavior of lubricants was tested in the boundary lubrication regime using a reciprocating ball-on-flat configuration (modified ASTM G 133 [26]) on a Plint TE 77 tribometer. About 10–15 mL of lubricant was used in each test and the contact interface was submerged. An M2 tool steel bar with a hardness of HRC 60 and a diameter of 25.4 mm was obtained from McMaster-Carr. The bar was machined into discs with a thickness of 3.2 mm and with the working surface polished to a roughness of 60–70 nm (*R*_a_) to serve as the flat specimens. An AISI 52100 bearing steel ball with a diameter of 10 mm, also obtained from McMaster-Carr, was used as the counterface sliding against the M2 steel disc. The roughness of the bearing ball was 25–50 nm (*R*_a_). To eliminate contaminants, both surfaces were thoroughly cleaned with isopropanol prior to testing. A constant load of 100 N was applied, corresponding to an initial Hertzian contact pressure of approximately 2.1 GPa. The tests were conducted with an oscillation frequency of 10 Hz and a sliding stroke of 10 mm. Each test lasted for a sliding distance of 1000 m under a temperature of 180 °F (82 °C), which is a typical operating temperature of hydraulic fluids. In these testing conditions, the central lubricant film thickness at the beginning of the test is less than 20 nm, as calculated using the Hamrock–Dowson formula [27]. The composite roughness of the ball and flat ($\sqrt{R_{q,ball}^{2}+R_{q,flat}^{2}}$) is above 100 nm. Therefore, the ratio of the lubrication film thickness to the composite roughness, known as the *λ* ratio, is less than 1, indicating boundary lubrication [27]. At least 2 repeat tests were conducted for each lubricant under the same conditions.

### 2.5. Surface Characterization

After the tribological test, the residue oil was rinsed off with isopropanol and the ball wear volume was quantified using a 3D white light interferometer (Wyko NT9100). The disc wear was not measurable as the wear depth was at the same level as the surface roughness. The ball wear scar morphology and composition were investigated using scanning electron microscopy (SEM) and energy-dispersive spectroscopy (EDS) on a Hitachi S4800 SEM equipped with an EDAX system. The EDS analysis was performed at a potential of 5.0 kV over a constant time of 100 sec. A cross-sectional scanning transmission electron microscopy (STEM) and EDS examination were conducted to reveal the thickness, nanostructure, and composition of the tribofilm formed by the best-performing IL. A Hitachi NB5000 gallium-focused ion beam (FIB) was used to lift the thin cross-section along the sliding direction from the worn surface of the AISI 52100 steel ball. A thin layer of carbon followed by tungsten was deposited on the selected area to protect the surface from ion-beam damage during the FIB process. After lifting, the FIB lamella was further thinned down (thickness < 100 nm) for the TEM analysis. The FIB process is illustrated step-by-step in Figure S2 in the Supporting Information. The thinned cross-section was analyzed using a JEOL JEM 2100 scanning transmission electron microscope equipped with an Oxford X-MAX Silicon Drift EDS Detector. The EDS data were analyzed using AZtec (version 3.3) software.

### 2.6. Toxicity Test

Only the ILs and commercial additives that demonstrated effective wear protection in the tribological tests were selected for the aquatic toxicity tests. The evaluation was performed in the Aquatic Ecology Laboratory at ORNL and followed a standard EPA protocol [28]. A sensitive model organism, the zooplankton *Ceriodaphnia dubia*, was employed for the toxicity testing. Zooplankton species have been used to estimate the aquatic toxicity of wastewaters and chemicals for decades [29]. Along with their wide environmental presence, *C. dubia* and related species offer many advantages, such as their relative ease of maintaining cultures in the lab (due to their small size and short life cycle (<30 days)), sensitivity to water quality, and high fecundity and parthenogenetic reproduction, all of which make them one of the most commonly used model organisms in biological research [30]. The OECD recommends the utilization of *Daphnia* and related species in standard tests to estimate aquatic toxicity [31].

**Figure 1:**
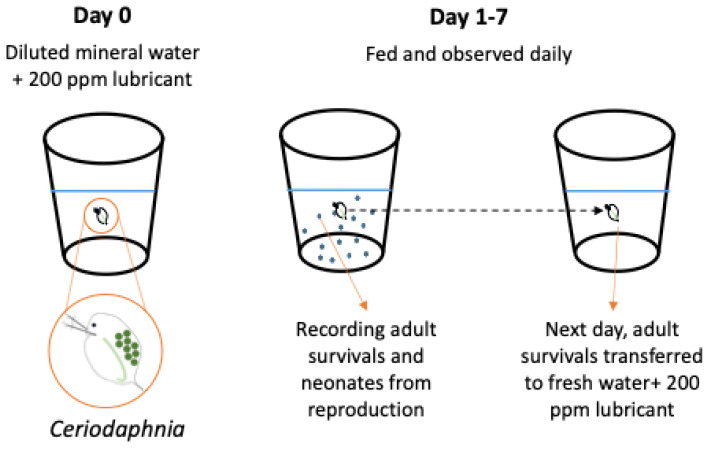
A 7–day aquatic chronic toxicity test using an EPA protocol [28].

The *C. dubia* used in these experiments were all laboratory-bred neonates (<24 h old) born within 8 h of each other, as defined by the EPA method. A 7-day test was conducted in diluted mineral water (DMW [28]). As illustrated in Figure 1, each individual *C. dubia* was placed in 15 mL of the treated solution and housed in an incubator under a 16:8 diurnal cycle at 25.0 °C. The *C. dubia* were fed and observed daily, with data on survival and reproduction recorded and reported in comparison to control treatments for each test. Preliminary tests were carried out with 200 ppm of the BT-mix base oil containing 0.5 wt% of the four ILs and the commercial Irgalube-349. The lubricant concentration of 200 ppm was chosen because EPA acute toxicity categorizes a substance as “Not Toxic” if its 48-h half-maximal effective concentration (EC50) is above 100 ppm. The initial 0.5 wt% additive concentration in the BT-mix was chosen because 0.5 wt% is typical for anti-wear additives in hydraulic fluids and it was used in the tribological testing. There were no statistically significant differences among the tested lubricants. The *C. dubia* had 100% survival rates for all four oils containing ILs and a 90% survival rate for the oil containing Irgalube-349 after 7 days. To better distinguish the additives, the concentration of additives was increased by 5 times to 2.5 wt% in the BT-mix and the lubricant concentration remained at 200 ppm in the test water. If an additive is categorized as “Not Toxic” at 2.5 wt%, it can be claimed as “Not Toxic” at any concentration equal to or less than 2.5 wt%.

## 3. Result and Discussion

### 3.1. Molecular Structures, Thermal Stability, and Oil Solubility of the ILs

**Figure 2:**
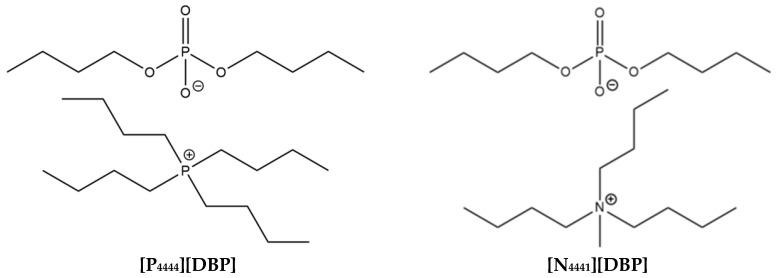

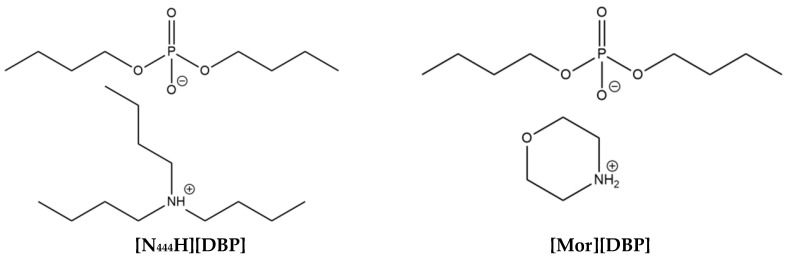
Molecular structures of the candidate ILs.

**Figure 3:**
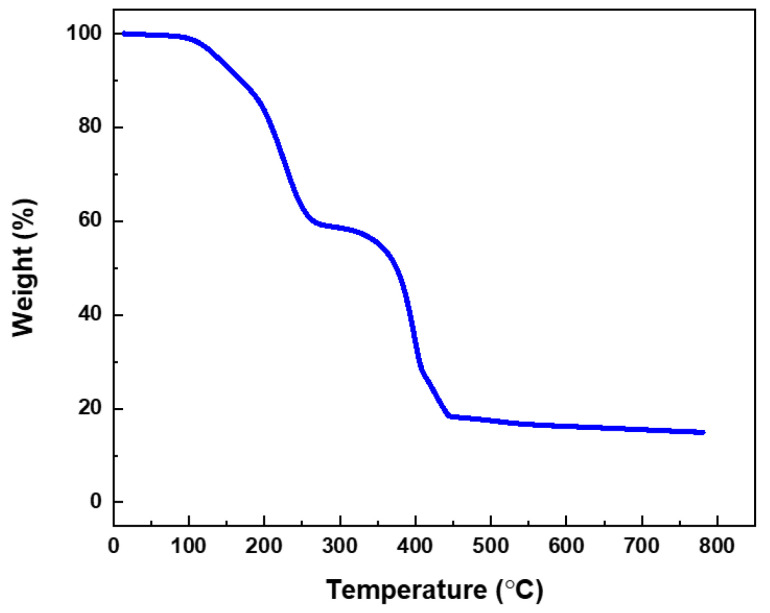
TGA profile of [P_4444_][DBP].

In previous work, the eco-friendly molecular design strategy was discussed in detail [9]. The molecular structures of the four ILs synthesized and studied in this work are displayed in Figure 2. The TGA profile of the phosphonium-phosphate, [P_4444_][DBP], is shown in Figure 3, while the TGA results of the other three ILs can be found in our earlier report [9]. The onset decomposition temperatures of 10% weight loss of [P_4444_][DBP], [N_4441_][DBP], [N_444_H][DBP], and [Mor][DBP] in the air environment are around 175, 210, 85, and 150 °C, respectively. Most candidate ILs and commercial anti-wear additives appear to possess a solubility of >5 wt% in the BT-mix base oil, except for [P_4444_][DBP], which has an oil solubility of 1–2 wt%, as shown in Table 2.

### 3.2. Tribological Behavior

**Figure 4:**
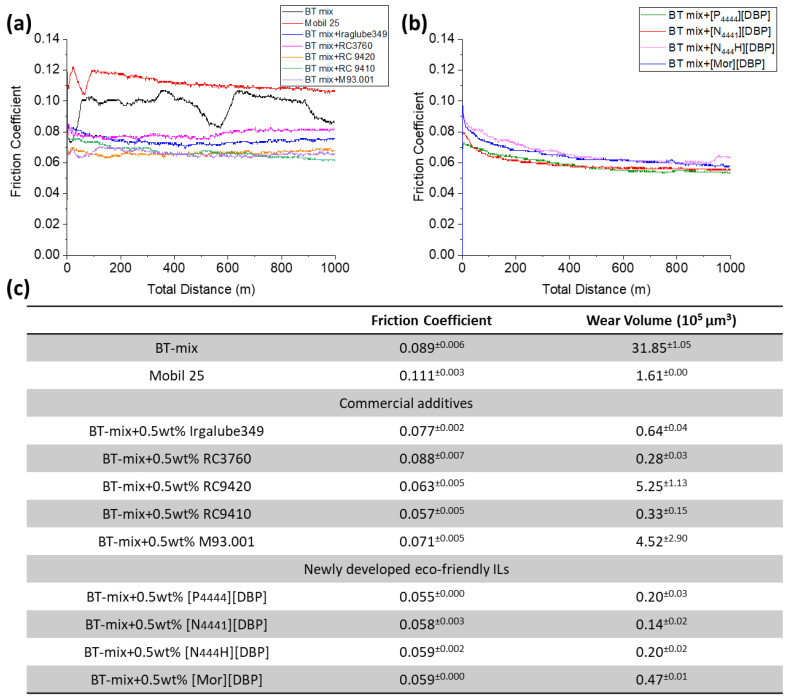
Tribological results: (**a**) friction traces of a fully formulated hydraulic fluid, the neat base oil, and the base oil containing commercial anti-wear additives; (**b**) friction traces of the base oil containing ILs; and (**c**) a summary of the average friction coefficients and wear volumes of all test lubricants. All lubricants had the same viscosity grade, VG 46, and all additives were at the same concentration of 0.5 wt%.

Figure 4 displays the friction and wear results obtained for the fully formulated Mobil 25 hydraulic fluid, the BT-mix base oil (neat), and the BT-mix oil containing various additives at 0.5 wt%. Note that only the ball wear is reported here as the disc wear was not measurable because the wear depth was at the same level as the roughness. The additives at such a low concentration have proven to have minimal impact on the oil viscosity and all the test lubricants have the same viscosity grade of VG46. This allowed a side-by-side comparison between the four ILs and the five ashless commercial anti-wear additives at the same 0.5 wt% concentration. 

The neat BT-mix base oil exhibited a fluctuating friction coefficient between 0.08 and 0.11 (Figure 4a) and a high ball wear loss (~32 × 10^5^ μm^3^) due to the absence of a wear-protecting agent (Figure 4c). The friction fluctuation is suspected to be caused by repeated formation and delamination of an oxide film at the contact interface. The fully formulated Mobil 25 oil displayed a slightly higher but more stable friction coefficient (~0.11). However, the ball wear volume was significantly reduced to ~1.6 × 10^5^ µm^3^. Mobil 25 oil likely contains ZDDP, the most common anti-wear additive in hydraulic fluids, which could be responsible for the good wear reduction and more stable but higher friction coefficient. This is because ZDDP is known to form a high-friction, wear-protective patch-like tribofilm [8,32].

The addition of the traditional ashless Irgalube-349 or a bio-derived product led to a reduction in the friction coefficient to a range of 0.5–0.8, with the exception of RC3760, which appeared to be ineffective in reducing friction. In terms of wear, RC9420 and M93.001 showed a lower ball wear value (4.5~6.5 × 10^5^ µm^3^) compared with the neat base oil but was significantly worse than Mobil 25. On the other hand, three other products, Irgalube-349, RC3760, and RC9410, were able to decrease the wear volume to 0.64, 0.28, and 0.33 × 10^5^ µm^3^, which was even better than the fully formulated Mobil 25, as shown in Figure 4c. 

The friction traces of the BT-mix base oil containing ILs are presented in Figure 4b. It is worth noting that the friction coefficient remained consistently low and stable within a narrow range of 0.55 to 0.60. The ball wear losses in the four IL-containing oils were 0.20, 0.14, 0.20, and 0.47 × 10^5^ µm^3^ for [P_4444_][DBP], [N_4441_][DBP], [N_444_H][DBP], and [Mor][DBP], respectively. From the friction and wear perspective, the four ILs are at least on par with or outperform the commercial anti-wear additives.

**Figure 5:**
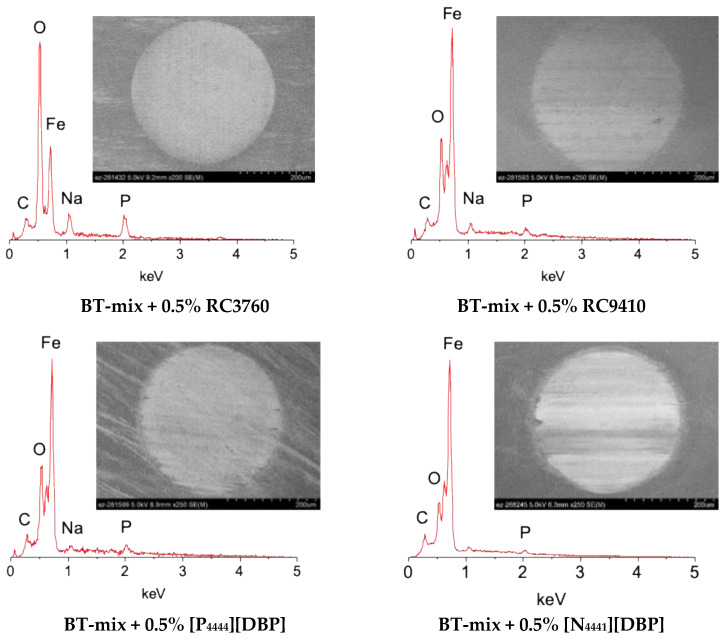

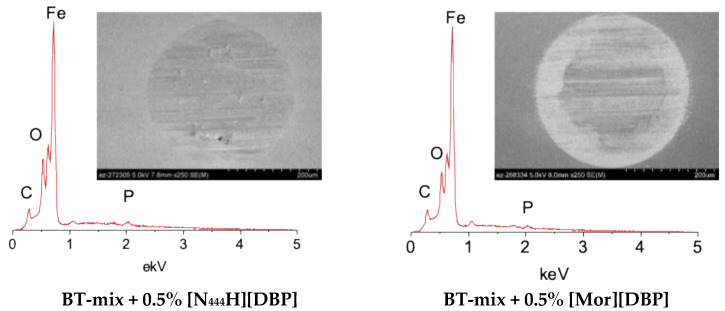
Worn surface SEM images and EDS spectra of the steel balls tested with the four ILs and the two top-performing commercial bio-derived additives.

The morphology and composition of the ball wear scars created by the four ILs are compared with those by the two most wear-protective commercial additives, RC3760 and RC9410, as shown in Figure 5. All six wear scars appear to be relatively smooth and uniform. All tribofilms seem to contain oxygen and phosphorus, suggesting a mixture of iron oxides and iron phosphates. Note that there is a sodium peak on all surfaces, which is particularly stronger on the two surfaces lubricated by the bio-derived additives. The source of sodium was not identified but it is suspected to be from either the chemical synthesis or sample handling.

**Figure 6:**
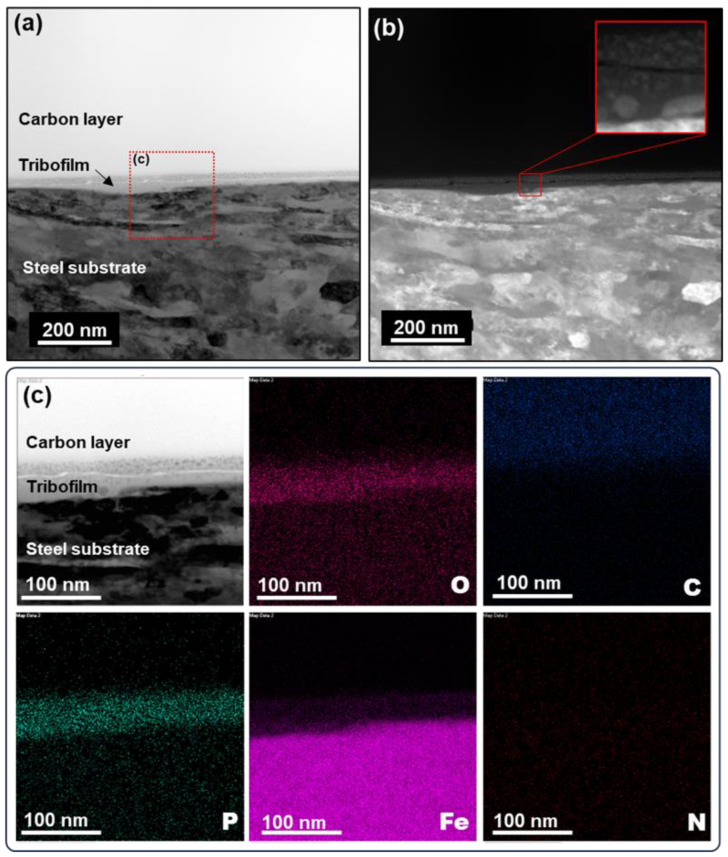
Cross-sectional STEM images and EDS elemental maps of the tribofilm formed on the steel ball tested with BT-mix + 0.5% [P_4444_][DBP]. (**a**) STEM bright field image, (**b**) STEM dark field image, and (**c**) EDS elemental maps.

The RC3760 tribofilm shows significantly higher peaks of oxygen and phosphorus compared with the rest, and the RC9410 tribofilm contains a small amount of sulfur that was not detected in the other tribofilms. The tribofilm from the phosphonium-based [P_4444_][DBP] seems to have a higher phosphorus content compared with the other three tribofilms from the ammonium-based ILs. The nanostructure and compositional maps of the [N_4441_][DBP] tribofilm were revealed in our previous report [9]. Here, the [P_4444_][DBP] tribofilm was further investigated, and Figure 6 displays the cross-sectional bright field scanning transmission electron microscopy (STEM) images and EDS elemental maps. The tribofilm looks like it consists of two layers with a total thickness ranging from 35 to 50 nm across the FIB-lifted 10-μm wide section (see Figure 6a). Embedded within the tribofilm are nano-sized particles, possibly wear debris, which are more concentrated in the tribofilm’s upper layer, as shown in Figure 6b. These particles are depicted as dark regions in the bright field image (Figure 6a) and as bright regions in the dark field image (Figure 6b). The top surface of the tribofilm appears to be smoother than the substrate, correlating to the low friction behavior (see Figure 4b). The elemental mapping in Figure 6c suggests that the tribofilm contains Fe, O, and P, where Fe originates from the steel substrate, O results from the rubbing-induced oxidation, and P is contributed from the IL.

The general IL tribofilm formation mechanism was discussed in detail in previous work based on the longer-chain phosphonium-phosphate ILs [P_66614_][DEHP] [33] and [P_8888_][DEHP] [34], and a multi-step process was proposed, including direct surface reactions followed by mechanical and chemical depositions of tribochemical reaction products between wear debris and reactive elements. The tribofilms formed by [P_66614_][DEHP] [33,35] and [P_8888_][DEHP] [34,36] were reported with similar nanostructures and compositions to that of [P_4444_][DBP], but appeared to be thicker (100–200 nm). The tribofilm thickness comparison based on STEM images may be unreliable because the very small scope (a few μm) may not be representative. On the other hand, one could argue that longer-chain phosphates might be easier to cross-link and polymerize [37], leading to a thicker tribofilm. However, it has been repeatedly reported that there is no correlation between tribofilm thickness and tribological performance [32,37]. Considering the relevance of the chemistry and molecular structure between [P_4444_][DBP] and the longer-chain phosphonium-phosphate ILs as well as the similarities in the nanostructure and composition of their tribofilms, the [P_4444_][DBP] tribofilm is likely formed by a similar process and composed of a mixture of iron oxides and iron phosphates, as described in [33,34].

### 3.3. Aquatic Toxicity 

The seven additives with superior wear protection, including the four ILs and the three commercial additives, Irgalube-349, Additin RC3760, and Additin RC9410, were selected for the aquatic toxicity evaluation. All additives were blended into the BT-mix base oil at 2.5 wt% and each lubricant was added to the test water (DMW) at 200 ppm in the aquatic toxicity tests.

**Figure 7:**
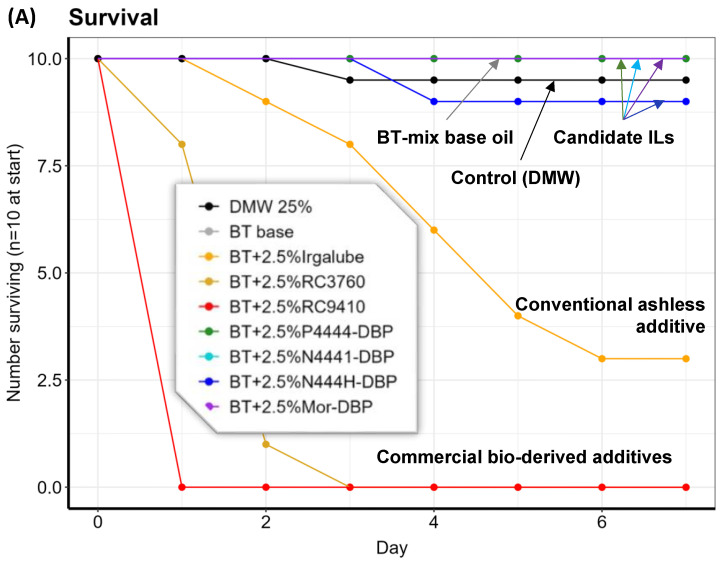

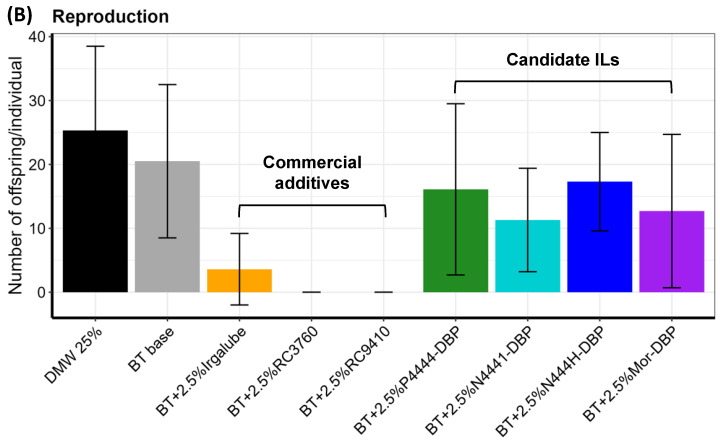
*C. dubia* survival (**A**) and reproduction (**B**) when exposed to 200 ppm of each lubricant in DMW media.

The *C. dubia* survival and reproduction results from the seven-day chronic toxicity tests are presented in Figure 7 below and in Table S2 in the Supporting Information. The control group underwent testing in DMW using the same procedure. The 200 ppm of neat BT-mix oil produced 100% survival of the *C. dubia* and 25 ± 8.2 neonates per survival, nearly identical to the DMW control. In contrast, the survival rate of *C. dubia* in the Irgalube-349-treated solution began to decline from day 2, with only three survivors by the end of the tests and the number of neonates at 14% compared with the control. Surprisingly, the two bio-derived additives, RC3760 and RC9410, appeared to be more toxic than the Irgalube-349 and caused the death of all *C. dubia* after three days and one day of the experiments without any reproduction. This is a clear example that bio-derived does not mean low toxicity. 

The four ILs demonstrated much better compatibility with the *C. dubia*. [P_4444_][DBP], [N_4441_][[DBP], and [Mor][DBP] exhibited 100% survival rates (their survival curves fully overlapped each other, as shown in Figure 7A) and [N_444_H][DBP] had a 90% survival rate. The acute toxicity categories for the aquatic environment are defined as “Very Toxic” (≤1 mg/L), “Toxic” (>1 mg/L but ≤10 mg/L), “Harmful” (>10 mg/L but ≤100 mg/L), and “Not Toxic” (>100 mg/L), based on the 48-h EC50 of crustacea [28]. Since the lubricants were tested at 200 ppm, these results indicate that the four ILs can be classified as “Not Toxic” when used as lubricant additives at a concentration of no more than 2.5 wt%. 

It should be noted that such a “Not Toxic” categorization does not mean no negative impact. For example, the *C. dubia*’s reproduction was reduced to various extents, from 56% to 68% of control reproduction, when exposed to [N_4441_][[DBP], [N_444_H][DBP], or [Mor][DBP]. In contrast, the reduction in reproduction by [P_4444_][DBP] was statistically insignificant: 23 ± 9.3 offspring per surviving individual vs. 25.3 ± 13.3 for the control. Nevertheless, the four ILs demonstrated distinctly lower toxicity compared with the traditional ashless and two commercial bio-derived additives. 

### 3.4. ILs vs. Commercial Anti-Wear Additives 

By collectively considering the lubricity and aquatic toxicity results presented above, it is evident that the selected short-chain ILs surpassed the commercial anti-wear additives, including the traditional ashless and bio-derived additives. The four ILs demonstrated superior friction- and wear-reducing performance to the commercial anti-wear additives and, even more impressively, they showed significantly lower toxicity than the commercial products and can be categorized as “Not Toxic”. Among these candidates, [P_4444_][DBP] stood out. In terms of toxicity, [P_4444_][DBP] led to a 100% survival rate and its reproduction rate closely matched that of the control group. Additionally, its tribological performance was among the top performers.

## 4. Conclusions

In summary, this study investigated the tribological performance and aquatic toxicity of four short-chain ILs, phosphonium-phosphate, aprotic ammonium-phosphate, protic ammonium-phosphate, and cyclic ammonium-phosphate, as candidate additives for EALs. The tribological and toxicity results were benchmarked against those of a conventional ashless and four commercial bio-derived anti-wear additives. Ball-on-flat reciprocating sliding boundary lubrication tests were conducted using a synthetic ester (BT-mix) base oil containing the ILs and commercial additives at the same 0.5 wt% concentration. The four ILs demonstrated a 30–40% friction reduction and a >99% wear reduction compared with the neat base oil, which was at least on par with or even better than the best performer of the five commercial additives. An EPA standard chronic aquatic toxicity test was carried out on the four ILs and three selected commercial additives that had shown good lubricating performance. The *C. dubia* had 90–100% survival when exposed to 200 ppm of the BT-mix oil containing 2.5 wt% ILs, but the survival rates dropped to 30% for the conventional ashless additive and 0% for the two commercial bio-derived additives at the same concentration. Among the selected ILs, [P_4444_][DBP] emerged as the most promising candidate with the lowest friction, second lowest wear, and lowest toxicity.

## Figures and Tables

**Table 1:** Calculation of the base oil mixing based on the viscosity.

	Mobil 25 Fully Formulated Hydraulic Oil	BT-4 Base Oil	BT-22 Base Oil	BT-Mix (Blend of BT-4 and BT-22 @ 0.593:0.407 Mass Ratio)
Viscosity @ 40 °C (cSt)	46	23.2	159.5	44.8
Viscosity @ 100 °C (cSt)	7.1	4.4~4.9	23.9	6.7
Biodegradability (OECD 301B)	-	88%	79%	-
EcoToxicity (OECD 201/202/203)	-	>1000 mg/L (SDS)	>1000 mg/L (SDS)	-

**Table 2:** Solubility of the additives in the BT-mix base oil.

Ionic Liquid	Solubility in BT-Mix	Commercial Additive	Solubility in BT-Mix
[P_4444_][DBP]	1–2 wt%	Irgalube-349	>5 wt%
[N_4441_][DBP]	>5 wt%	RC3760	>5 wt%
[N_444_H][DBP]	>5 wt%	RC9420	>5 wt%
[Mor][DBP]	>5 wt%	RC9410	>5 wt%
		M93.001	>5 wt%

## Data Availability

This manuscript has been authored by UT-Battelle, LLC, under contract DE-AC05-00OR22725 with the U.S. Department of Energy (DOE). The U.S. government retains and the publisher, by accepting the article for publication, acknowledges that the U.S. government retains a nonexclusive, paid-up, irrevocable, worldwide license to publish or reproduce the published form of this manuscript, or allow others to do so, for U.S. government purposes. The U.S. DOE will provide public access to these results of federally sponsored research in accordance with the DOE Public Access Plan (http://energy.gov/downloads/doe-public-access-plan, accessed on 11 August 2024).

## Supplementary Materials

The following supporting information can be downloaded at: https://www.mdpi.com/article/10.3390/molecules29163851/s1.
