# Supplementary material for: Comparison of Eco-Friendly Ionic Liquids and Commercial Bio-Derived Lubricant Additives in Terms of Tribological Performance and Aquatic Toxicity
Source: Molecules. 2024 Aug 14;29(16):3851. doi: 10.3390/molecules29163851 (PMC11357439; doi:10.3390/molecules29163851)
Supplement: Supplementary file 1 [file molecules-29-03851-s001.zip › molecules-3133331-supplementary.pdf]

Supporting Information

Comparison of Eco-Friendly Ionic Liquids and Commercial Bio-Derived Lubricant Additives in Terms of Tribological Performance and Aquatic Toxicity

Xin He [1,*,†], Louise M. Stevenson [2], Chanaka Kumara [1], Teresa J. Mathews [2], Huimin Luo [3] and Jun Qu [1,*]

[1] Materials Science & Technology Division, Oak Ridge National Laboratory, Oak Ridge, TN 37831, USA; kumarack@ornl.gov
[2] Environmental Sciences Division, Oak Ridge National Laboratory, Oak Ridge, TN 37831, USA; stevensonlm@ornl.gov (L.M.S.); mathewstj@ornl.gov (T.J.M.)
[3] Manufacturing Science Division, Oak Ridge National Laboratory, Oak Ridge, TN 37831, USA; luoh@ornl.gov
* Correspondence: xin.he@syensqo.com (X.H.); qujn@ornl.gov (J.Q.)
† Current address: Syensqo, Bristol, PA 19007, USA.

Number of Tables: 2

Number of Figures: 2

Table S1. Composition of the commercial anti-wear additives based on the safety data sheets.

Anti-wear additive (Company)	Nominal composition (according to SDS)
Irgalube 349 (BASF)	- Amines, C11-14-branched alkyl, monohexyl and dihexyl phosphates (>=90-100%)
ADDITIN M 93.001 (LANXESS)	- 2,6-di-tert-butyl-p-cresol (1-5%)<br>- 1H-Benzotriazole-1-methanamine, N,N-bis(2-ethylhexyl)-ar-methyl- (1-5%)<br>- Amines, C11-14-branched alkyl, monohexyl and dihexyl phosphates (1-5%)<br>- Butanedioic acid, 2-(tetrapropenyl)-, ester with 1,2-propanediol (1-5%)<br>- Distillates (petroleum), hydrotreated light naphthenic (1-5%)<br>- (tetrapropenyl)succinic acid (1-5%)<br>- Mineral oil (~1%)
ADDITIN RC 3760 (LANXESS)	- Amines, C11-14-branched alkyl, monohexyl and dihexyl phosphates (100%)
ADDITIN RC 9410 (LANXESS)	- Combination of phosphorus-sulfur compounds with oxidation and corrosion inhibitors<br>- Mineral oil (~20%)
ADDITIN RC 9420 (LANXESS)	- Distillates (petroleum), solvent-dewaxed heavy paraffinic (20-30%)<br>- Amines, C11-14 Branched Alkyl Monohexyl and dihexyl phosphates (10-20%)<br>- Aliphatic dibasic acid, glycol ester (1-3%)<br>- Distillates, petroleum, hydrotreated light naphthenic (1-5%)<br>- Tetrapropenyl Succinic Acid (1-5%)<br>- Benzotriazole (1-5%)<br>- Mineral oil (~30%)

Table S2. C. dubia survival and reproduction results of a 7-day aquatic chronic toxicity test using an EPA protocol

**Lubricant** @ 200 ppm in the test water		# of *C. dubia* surviving[1]				Survival %	Avg. neonates per surviving adult
		Day 1	Day 2	Day 3	Day 7		
Dilute mineral water (control, two repeats)		10	10	9-10	9-10	95%	25.3 ± 13.3
Neat BT-mix base oil (without additives)		10	10	10	10	100%	25.0 ± 8.2
Commercial additives	BT-mix+ 2.5% Irgalube-349	10	9	8	3	30%	3.6 ± 5.6
	BT-mix+ 2.5% RC3760	8	1	0	0	0%	0
	BT-mix+ 2.5% RC9410	0	0	0	0	0%	0
Candidate ionic liquids	BT-mix+ 2.5% [P_{4444}][DBP]	10	10	10	10	100%	23.0 ± 9.3
	BT-mix+ 2.5% [N_{4441}][DBP]	10	10	10	10	100%	14.1 ± 6.3
	BT-mix+ 2.5% [N_{444}H][DBP]	10	10	10	9	90%	17.3 ± 7.7
	BT-mix+ 2.5% [Mor][DBP]	10	10	10	10	100%	15.9 ± 11.2

[1]*All treatments started with 10 individuals*

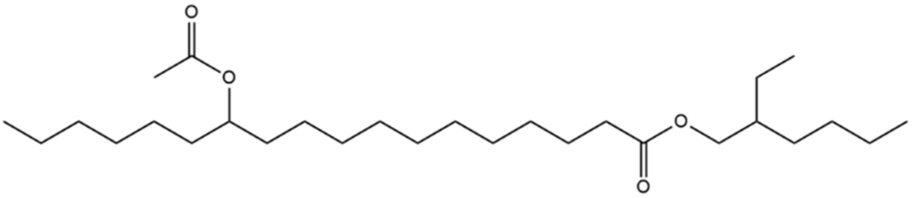

BT-4

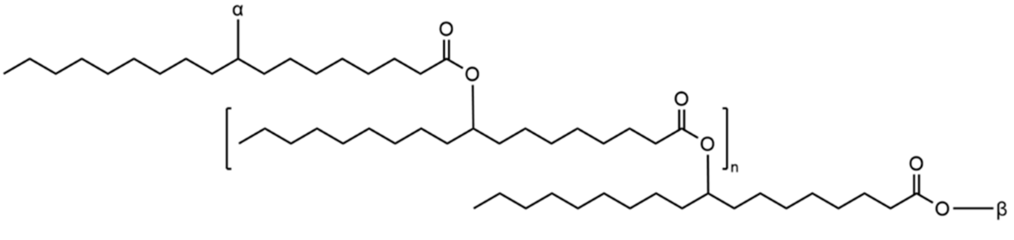

BT-22

Figure S1. Molecular structures of the two base oils.

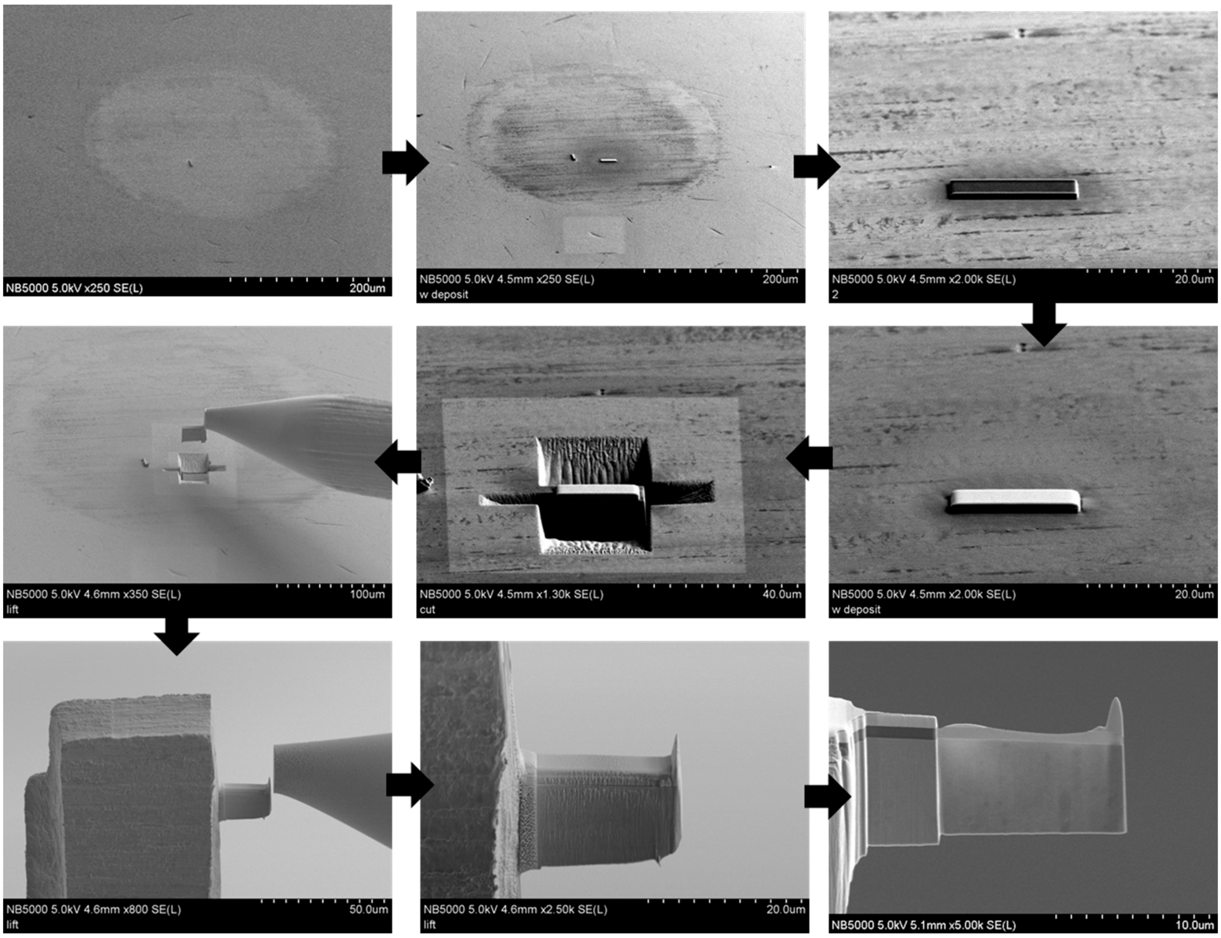

Figure S2. FIB process lifting a cross-section from the wear scar and then thinning for STEM/EDS.
